# Roles of vacuum tunnelling and contact mechanics in single-molecule thermopower

**DOI:** 10.1038/srep44276

**Published:** 2017-03-10

**Authors:** Makusu Tsutsui, Kazumichi Yokota, Takanori Morikawa, Masateru Taniguchi

**Affiliations:** 1 The Institute of Scientific and Industrial Research, Osaka University, 8-1 Mihogaoka, Ibaraki, Osaka 567-0047, Japan

## Abstract

Molecular junction is a chemically-defined nanostructure whose discrete electronic states are expected to render enhanced thermoelectric figure of merit suitable for energy-harvesting applications. Here, we report on geometrical dependence of thermoelectricity in metal-molecule-metal structures. We performed simultaneous measurements of the electrical conductance and thermovoltage of aromatic molecules having different anchoring groups at room temperature in vacuum. We elucidated the mutual contributions of vacuum tunnelling on thermoelectricity in the short molecular bridges. We also found stretching-induced thermoelectric voltage enhancement in thiol-linked single-molecule bridges along with absence of the pulling effects in diamine counterparts, thereby suggested that the electromechanical effect would be a rather universal phenomenon in Au-S anchored molecular junctions that undergo substantial metal-molecule contact elongation upon stretching. The present results provide a novel concept for molecular design to achieve high thermopower with single-molecule junctions.

Thermoelectricity is a phenomenon wherein a temperature difference in a material induces energy flow via electrical charge diffusion from hot to cold side thereby providing a simple and environmentally-friendly way of converting heat into electricity in single step without any need of moving mechanical components equipped in many of today’s thermal power generation systems^1^. Wide applications of this ideal convertor require compounds with high thermoelectric figure of merits^2^,^3^,^4^,^5^. Recently, there has been growing interest in exploring quantum confinement effects in low-dimensional structures for better thermoelectrics^6^,^7^,^8^,^9^,^10^,^11^. Alike the nanomaterials investigated to date, such as superlattices^12^, nanotube/nanowires^13^, and quantum dots^14^, a single-molecule interconnected to metallic electrodes is a quantum system having discrete electronic states that promise giant thermopower by chemically-engineering the electronic structures through optimizing the molecular architectures^15^,^16^,^17^,^18^. Much progress has been accomplished in understanding and controlling the single-molecule thermoelectric transport properties, such as length dependence^19^,^20^,^21^, intermolecular interactions^22^, and gate control^23^, owing to the advance in experimental techniques to address the electron and heat transport in molecular bridges^17^. In sharp contrast, the roles of electrode-molecule contacts on the thermoelectric properties have remained almost unexplored^24^,^25^ albeit the theoretically predicted impact on the electronic structures^26^,^27^,^28^,^29^,^30^, due mostly to the technical difficulty to evaluate the geometrical dependence of thermoelectricity in molecular junctions that requires a reliable method to form and hold a molecular bridge for long-enough time to measure their thermoelectric properties and meanwhile controllably deform the configurations at an atomistic level. In the present study, therefore, we report molecular junction diagnosis by thermoelectric analysis for characterizing the anchor dependence to geometrical sensitivity of single-molecule thermopower.

## Results

### Conductance and thermovoltage traces

**Figure 1:**
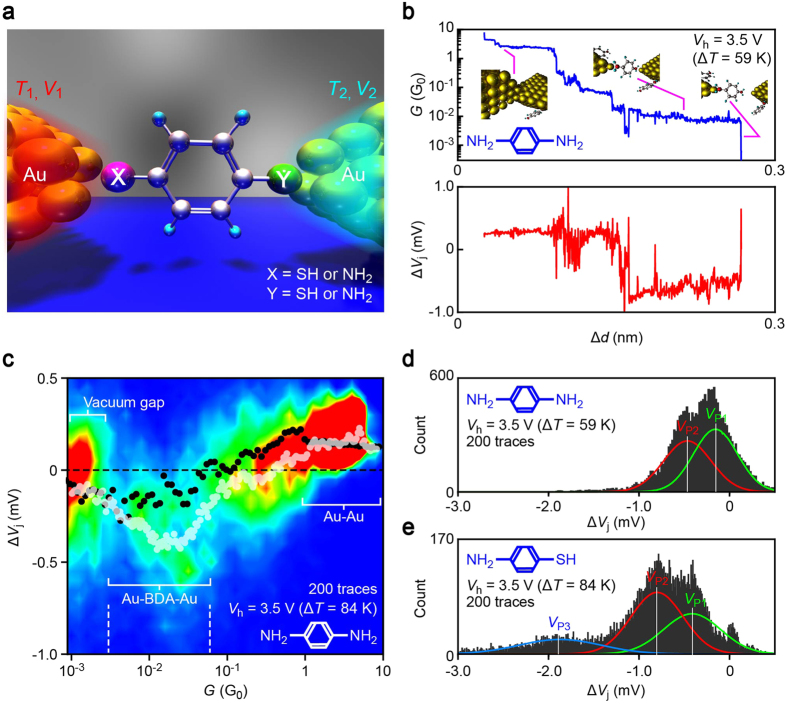
Thermoelectric measurements of molecular junctions. (**a)** Schematic model of single-molecule junctions comprised of a X-benzene-Y molecule bonded to two Au electrodes. Thermopower was measured by imposing a temperature differential through electrical heating of a microheater and probing the thermovoltage. X and Y tested were SH and/or NH_2_. (**b)** Conductance (*G*) and thermovoltage (Δ*V*_j_) traces recorded simultaneously during tensile breakdown of a Au-1,4-benzenediamine-Au bridge under the heater voltage *V*_h_ = 3.5 V displaying positive and negative thermovoltage of Au nanocontacts and the molecular junction, respectively. (**c)** *G* versus Δ*V*_j_ two-dimensional histogram. White and black plots are the average Δ*V*_j_ in cases with and without molecules being added to the junctions, respectively. (**d**,**e)** Δ*V*_j_ distributions constructed with the trace data showing *G* in a range from 6 × 10^−2^ *G*_0_ to 0.3 × 10^−3^ *G*_0_ for benzenediamine (BDA) (**d**) and aminobenzenethiol (ABT) (**e**). *V*_P1_, *V*_P2_, and *V*_P3_ denote the peak positions as defined by Gaussian fit to the histograms represented by solid curves.

We studied benzene molecules possessing amine and thiol groups at the *para* positions: 1,4-benzenedithiol (BDT), 1,4-benzenediamine (BDA), and 4-aminobenzenethiol (ABT) (Fig. 1a). Microheater-embedded lithographically-defined mechanically-controllable break junctions (MCBJs)^25^ were employed to carry out simultaneous measurements of the conductance *G* and the thermovoltage Δ*V*_j_ of metal-molecule-metal junctions under stretching at a rate of 0.5 pm/s through a piezo-voltage (*V*_piezo_) control with a temperature gradient imposed through Joule heat generated at the Pt coil with the applied voltage *V*_h_ at room temperature in vacuum (Figs S1 and S2; see also Fig. S9 for calibration measurements to convert *V*_piezo_ into the junction displacement Δ*d*). The breaking process was implemented for 200 times per *V*_h_ condition by reconnecting the Au contact after breakdown of molecular junctions. In each trial, a Au contact was broken at first to create a pair of nanoelectrodes, wherein *G* decreased to below 1 *G*_0_ in a discrete manner where *G*_0_ = 2*e*^2^/*h* is the conductance quantum with *e* and *h* denoting the electron charge and Planck constant, respectively (Fig. 1b). Thereafter, conductance plateaus were often found at 10^−2^ *G*_0_ to 10^−3^ *G*_0_ suggesting formations of molecular bridges^31^. Meanwhile, the concurrently recorded thermovoltage behaved somewhat synchronously to the change in the conductance (Fig. 1c). Specifically, positive Δ*V*_j_ was obtained at *G* > 1 *G*_α_ reflecting the negative thermopower *S*_Au_ = −Δ*V*_j_/(Δ*T*) of Au nanocontacts^32^, where Δ*T* is the temperature difference created therein (see Fig. S8 for estimations of Δ*T*), owing to the sign of the energy derivative of the density of electronic states at the Fermi level^33^. In contrast, the thermoelectric voltage turns out to be negative at the low conductance regime. The corresponding positive Seebeck coefficient implies charge transport through the highest-occupied molecular orbital (HOMO) levels for the molecules tested^34^,^35^.

### Conductance versus thermovoltage characteristics

In order to shed further light on molecular nature in the obtained thermoelectric properties, two-dimensional histograms of *G* and Δ*V*_j_ were constructed (Fig. 1c; see also Fig. S3). The plots conspicuously depict the presence of Au nanocontacts as a cluster of data accumulated at above 1 *G*_0_ with positive Δ*V*_j_. In contrast, the low conductance region below 1 × 10^−3^ *G*_0_ reveals a regime with negligibly small yet finite thermovoltage on average, which is attributable to tunnelling thermopower in a vacuum gap^36^,^37^ as corroborated by a control experiment wherein no molecules were added (black plots). On the other hand, there lies another distinct feature at the intermediate range of conductance around 10^−2^ *G*_0_ wherein the negative Δ*V*_j_ can be ascribed to the aforementioned HOMO-derived carrier transport in the molecular junctions with relatively large scattering presumably reflecting the geometrical sensitivity of the electronic structure^28^,^38^.

### Statistical variations of junction thermopower

**Figure 2:**
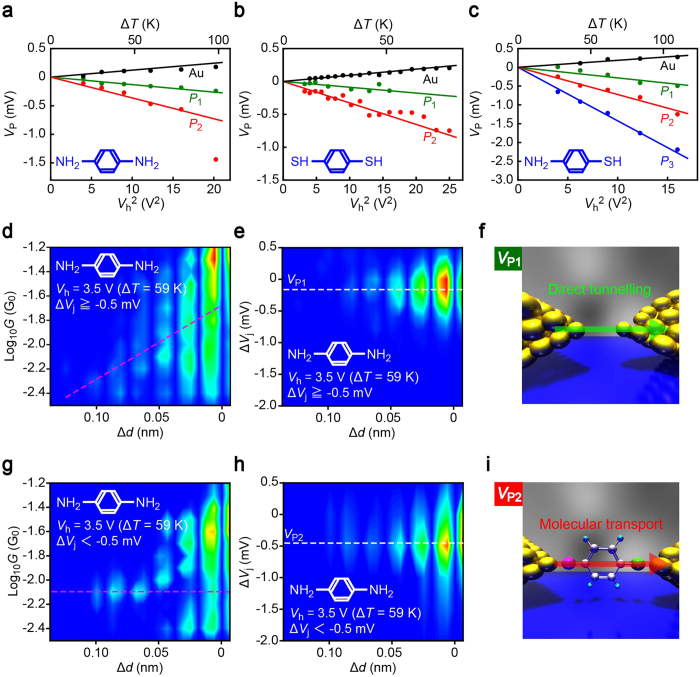
Molecular junction diagnosis. (**a–c)** Characteristic *V*_P_ states plotted as a function of *V*_h_^2^ and Δ*T* for BDA (**a**), BDT (**b**), and ABT (**c**). Solid curves are linear fitting to the plots. (**d–f**) Change in *G* (**d**) and Δ*V*_j_ (**e**) under mechanical elongation Δ*d* of Au-BDA-Au junctions showing Δ*V*_ave_ higher than −0.5 mV at Δ*T* = 59 K. The exponential decay in *G* indicates absence of molecules in the electrode gap in case for the thermopower states of *V*_P1_ (**f**). (**g–i)** High-thermovoltage traces with Δ*V*_ave_ ≤ −0.5 mV (**h**) reveal a plateau at close to the single-molecule conductance (**g**) whereby suggesting electron tunnelling through Au-BDA-Au bridges for V_P2_. Broken lines in (**d**) and (**g**) show a linear fit to the semi-logarithmic plots and the single-molecule conductance state, respectively.

Having confirmed the identity of molecular bridges, statistical variations of the thermoelectric voltage was analysed by building Δ*V*_j_ histograms with data extracted using a conductance window from 3 × 10^−3^ *G*_0_ to 6 × 10^−2^ *G*_0_ whereat the characteristic negative thermoelectric voltage was detected (we note that the single-molecule conductance falls in this range of conductance^39^,^40^,^41^). Interestingly, we found bimodal and trimodal distributions for the symmetric (BDA and BDT) and asymmetric (ABT) molecules, respectively (Fig. 1d; see also Figs S4 and S5). Gaussian fit extracted peaks positioned at *V*_P1_ through *V*_P3_. Plots of *V*_P_, along with the average thermovoltage of Au nanocontacts deduced from Δ*V*_j_ at *G* > 1 *G*_0_, with respect to the heater voltage illuminated linear increase in the absolute values of the characteristic thermoelectric voltage with *V*_h_^2^ (Fig. 2a–c). This is interpreted as due to the fact that the temperature difference at the junction is produced through the power *V*_h_^2^/*R*_h_ (*R*_h_ is the resistance of the heater) dissipated locally at the Pt micro-coil that heats up one side of the Au leads via thermal conduction through the Al_2_O_3_ layer^25^.

### Thermovoltage signatures of molecular junctions

It is of importance to elucidate the origin of the multiple thermovoltage states revealed by the Δ*V*_j_ histograms. For this, we inspected the conductance traces of junctions having average thermovoltage Δ*V*_ave_ = 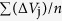 in the low conductance regimes suggestive of molecular bridge formations, where *n* is the number of data points in each Δ*V*_j_ − *t* curves (Fig. 2d–g). Interestingly, we found exponential decay in the conductance with respect to the distance *d*_gap_ between the Au electrodes in case of junctions with Δ*V*_ave_ ≥ −0.5 mV at *V*_h_ = 3.5 V (Δ*T* = 59 K), i.e. *V*_P1_ state, for BDAs (Fig. 2d–e), which is naturally ascribed to electron tunnelling through vacuum gaps instead of charge transport through molecular bridges (Fig. 2f)^42^. In sharp contrast, a plateau was observed at around 8 × 10^−3^ *G*_0_ for the plots with Δ*V*_ave_ < −0.5 mV (*V*_P2_ state) suggesting formations of Au-BDA-Au links (Fig. 2g–i)^40^. Similar trends were seen for the other molecules as well (see Fig. S6). These results imply that whereas *V*_P2_ is unequivocally attributed to the intrinsic thermoelectric transport characteristics of molecular junctions, *V*_P1_ is most likely the tunnelling thermopower of a vacuum gap, whose values are in fact close to the low thermovoltage detected for open contacts in the control experiments whereat no molecules are expected between the electrodes. Rough estimation of the barrier height energy Φ from the slope of the conductance decay by assuming *G* ∝ exp(−2*κd*_gap_) with the coefficient *κ* = 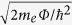 yields Φ = 6.8 eV with the electron mass *m*_e_ = 9.11 × 10^−31^ kg (Fig. 2d), which is close to the work function of Au^43^ thereby corroborating the vacuum tunnelling scenario for *V*_P1_.

### Stretching-induced thermopower enhancement

**Figure 3:**
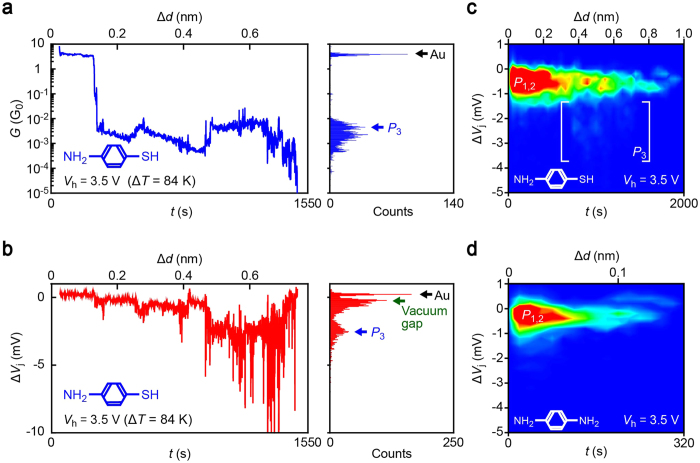
Stretching-induced thermopower enhancement. (**a,b)** *G* and Δ*V*_j_ curves demonstrating increased thermovoltage in a highly-elongated ABT junction. The histograms of the traces reveal the conductance and thermovoltage representative of the junction conditions at each stage of the tensile breakdown. The stretching caused *P*_3_ states with enhanced thermovoltage. (**c,d)** Two-dimensional Δ*V*_j_ histograms of ABT (**c**) and BDA junctions (**d**). Note that *P*_3_ states are present only in ABT.

The high thermovoltage states of *V*_P3_ observed for ABT (Fig. 1e) is a superior property from viewpoints of its thermoelectric applications compared to the *V*_P2_ states. We found that this distinct characteristics arises upon excessive elongation of the molecular junctions (Fig. 3a–d; see also Fig. S7). The stretching effects were also present in BDTs^25^,^26^ but were not evident in the thermovoltage histograms, and thus not considered here in detail due to the relatively short-lived nature of the high-Δ*V*_j_ states that emerges on the verge of mechanical breakdown, in comparison to the long lifetime of the Au-thiol linkages. On the other hand, the mechanically-induced thermopower enhancement was confirmed to be completely absent in BDAs indicating important roles of the thiol linkers (Fig. S3d).

## Discussion

### Direct tunnelling contributions

**Figure 4:**
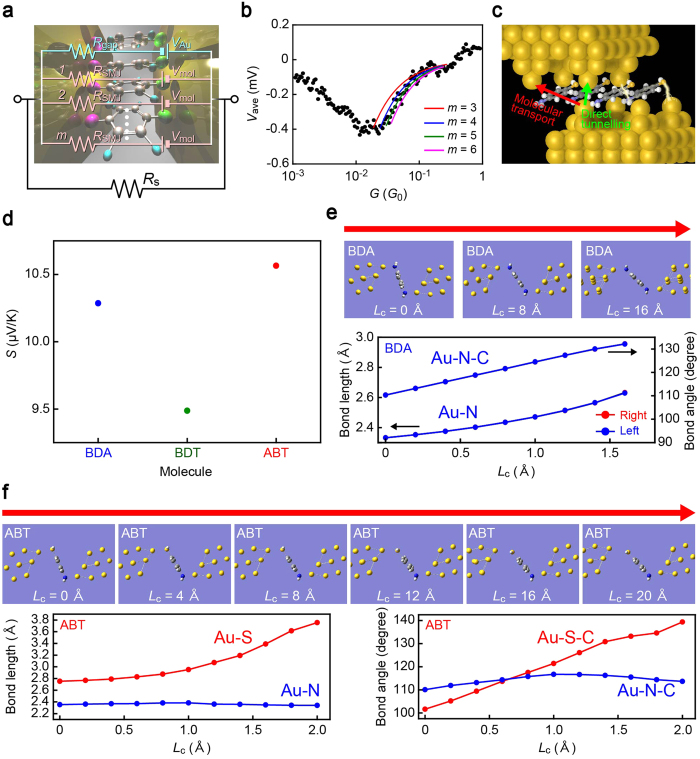
Anchor-dependent contact mechanics. (**a)** Equivalent circuit of molecular junctions consisting of *m* molecules with resistance *R*_SMJ_ and thermovoltage *V*_2_ bridging a Au electrode gap. Contribution of direct tunnelling is included as the resistance *R*_gap_ and the tunnelling thermopower component *V*_gap_ connected in parallel. *R*_s_ is the thermovoltage sensing resistor connected in series to the junctions. (**b)** Direct tunnelling contribution. The thermovoltage versus conductance characteristics complies with the circuit model assuming quadruple-molecule junctions with variable size of vacuum gap with 1/*R*_gap_ from 1 × 10^−4^ G_0_ to 1 × 10^−1^ G_0_. (**c)** Energy-optimized structure of BDA quadruple-molecule junctions. (**d)** Seebeck coefficients *S* estimated by taking the thermopower of Au nanocontacts as reference. (**e,f)** Contact mechanics of BDA (**e**) and ABT (**f**). Junction images show energy-optimized structures. Arrows point toward stretching of junctions defined as the distance *L*_c_ between the molecule-linked Au atoms. Au-molecule bond lengths and angles at the both sides are plotted with respect to *L*_c_.

From a naïve point of view, thermoelectric transport in molecular junctions should accompany an effect of direct tunnelling through vacuum^44^. In order to quantify its possible influence on the measured junction properties, we analysed the conductance versus thermovoltage characteristics in detail. First we noticed that although the positive Δ*V*_j_ at *G* > 1 *G*_0_ is suggestive of quantum thermopower of Au nanocontacts (Fig. 1c), a care should be taken on the quantitative interpretation as it is well-established that molecules are often bridging aside the Au atom-sized contacts before the opening of electrode nanogaps^45^,^46^. This can be seen as multiple batteries, each contributing the single-molecule thermovoltage, with internal resistance *R*_i_ connected in parallel when considering no notable quantum interference^47^,^48^ in the thermoelectric transport (Fig. 4a). Then, what we measured was actually the combined voltage described as Δ*V*_j_ = (*V*_Au_*mR*_mol_ + *V*_mol_*R*_Au_)(*R*_mol_*R*_Au_)/(*R*_mol_*R*_s_ + *R*_Au_*R*_s_ + *R*_mol_*R*_Au_)(*R*_mol_ + *R*_Au_), where *R*_mol_ = *R*_SMJ_/*m* is the net resistance of *m* molecules bridging in parallel with the single molecule resistance *R*_SMJ_, *R*_Au_ is the resistance of the Au contacts, and *R*_s_ is the serial resistance. This equation tells us that the junction thermovoltage is prone to be determined solely by the source, either molecules or tunnelling gaps, having lower resistance. As *R*_SMJ_ in the present study is in a range of MΩ^39^,^40^,^41^, *V*_mol_ virtually vanishes and Δ*V*_j_ is dominated by that engendered at the Au contacts whereby yielding the positive thermovoltage of the atomic chains at the conductance above 1 *G*_0_. In the meantime, it rapidly decreases to a small negative value as *G* declines to below 1 *G*_0_, which indicates opening of an electrode gap wherein a few molecules can be connected to the electrodes. Whether molecular feature becomes observable at this stage is thus determined by the relative significance of the thermopower contributions of molecular bridges and vacuum gaps^47^. On the other hand, we find nice fit on the Δ*V*_j_ − *G* characteristics with the parallel circuit model by assuming four molecules bridging the electrodes (*m* = 4) with *R*_SMJ_ = 2.2 MΩ and *V*_P2_ = *V*_mol_ = 0.47 mV and variable tunnelling gap size having conductance from 0.1 × 10^−3^ *G*_0_ to 0.2 *G*_0_ with *V*_gap_ = *V*_P1_ = 0.054 mV at *V*_h_ = 3.5 V (Δ*T* = 59 K) for BDA junctions (Fig. 4b), thus elucidating the non-negligible influence of vacuum tunnelling on the molecular junction thermoelectric transport (Fig. 4c). This in turn suggests the possible use of thermopower for molecular junction diagnosis to assess the vacuum gap contribution as well as to count the number of energy-carrying molecules, which, in the present work, manifested the importance to widen inter-electrode distance to mitigate the detrimental influence of direct tunnelling on the thermoelectric performance of single-molecule junctions.

### Anchor dependence of molecular thermopower

Quantitative evaluations of molecular junction thermopower involves intriguing argument about the temperature differential in the atomic scale systems^49^. Here, we instead examined a comparative analysis of the anchor dependence of the molecular junction thermopower by exploiting the Au junctions as an atomic thermometer to characterize the temperature differential. The slope *α* of the linear *V*_Au_ − *V*_h_^2^ dependence constitutes a well-defined Seebeck coefficient of the metal nanocontacts *S*_Au_ = −*α*_Au_/*β* probed in the present measurements, where *β* is a coefficient that converts the heater voltage to the temperature difference as Δ*T* = *βV*_h_^2^. Here, *S*_Au_ contains a bulk contribution of *S*_bulk_ = 1.96 μV/K^50^ in addition to the quantum thermopower *S*_q_ at the atom-sized contacts as they were connected to millimeter-long leads. We calculated *β* by comparing *S*_Au_ = *S*_q_ − *S*_bulk_ to the experimental −*α*_Au_ with *S*_q_ = −0.75 μV/K for ballistic Au atom-sized contacts^33^ whose radius *r*_c_ is much smaller than the electron mean free path *l* in the conductance range of 1 *G*_0_ < *G* < 5 *G*_0_. This yielded *S*_BDT_ = 9.5 μV/K for *V*_P2_ of Au-BDT-Au bridges, which fairly agrees with the previous literatures^33^. Comparatively, BDA and ABT exhibited higher thermoelectric power of *S*_ABT_ = 10.6 μV/K and *S*_BDA_ = 10.3 μV/K, respectively (Fig. 4d).

### Geometrical dependence of molecular junction thermopower

We have observed a concomitant enhancement of the conductance and thermopower in highly-stretched thiol-anchored molecular junctions. We explored the underlying contact mechanics by first-principles calculations of junction motifs under various inter-electrode distance conditions *L*_c_, which is defined as the displacement of the molecule-linked Au atoms from the optimized configurations, to unveil the cause of stretching-induced thermopower enhancement observed for the thiolate molecules. We found that while BDAs cannot withstand the tensile straining above 1.6 Å (Fig. 4e), due to the restricted Au-NH_2_ bond configurations that hinders the conformational degrees of freedom of molecules in the electrode gap^51^, Au-S bond angles and lengths are more amenable to the mechanical stretching reflecting the diverse forms of covalent interactions between the sulphur and Au surface atoms (Fig. 4f)^52^, which allowed ABT to take upright conformations. Here, it is worth noting that the Au-S bond in ABT is capable of being elongated by up to 1 Å whereas the NH_2_-Au links can be pulled apart by less than 0.3 Å. Under this level of bond elongation, it is anticipated theoretically that *G* increases by junction stretching due to the entailed shift in the conducting molecular orbital level toward the electrode Fermi level *E*_F_^53^,^54^,^55^. In a framework of single-particle model, the mechanical modulation of molecular states also anticipates thermopower enhancement since the slope of transmission curve at *E*_F_ tends to be steeper as the transport becomes closer to a resonance, the overall trend of which explains why the mutual enhancement of *G* and Δ*V*_j_ was observed only in the thiol-anchored molecular junction, thereby suggesting in turn that the electromechanical characteristics would rather be a universal feature in stretched molecular junctions having at least one Au-S link^56^, the verification of which calls for future efforts to explore the mechanical effects on various organic molecules other than the mono-benzene derivatives studied in the present work. These findings provide a guide for designing optimal anchoring groups for achieving high thermoelectric power with single-molecule junctions.

## Methods

### Fabrication of heater-embedded MCBJs

Microelectrodes were formed on a polyimide coated phosphor bronze substrate by photolithography and radio-frequency magnetron sputtering of a Cr/Au (2 nm/50 nm thickness) layer followed by lift-off through sonication in *N,N*-dimethylformamide. Subsequently, Al_2_O_3_ thermal bath was created by electron beam lithography, inductively-coupled plasma sputter deposition of Al_2_O_3_ (40 nm thick), and the lift-off process. On the heat reservoir, Pt coils and a Au nanojunction were further prepared by two additional electron beam lithography processes. Finally, the polyimide layer was deep-etched by 2 μm in depth through reactive ion etching using O_2_ etchant gas so as to free the Au junction from the substrate. The free-standing length of the junction was approximately 2 μm, which provided the attenuation factor *r* of 1.3 × 10^−4^ as calibrated by measuring electrode gap size dependence of the tunnelling current (Fig. S9).

### Formations of molecular junctions

A heater-embedded MCBJ was mounted on a sample stage where there were two counter supports. On the other side, there was a piezo-driven pushing rod that expands or shrink by changing the voltage *V*_p_ by Δ*V*_p_ with ratio −1 μm/*V*_p_. In experiments, a droplet of dilute toluene solution of molecules (either BDA, BDT, or ABT) at concentration 5 μM was added on a Au junction. The MCBJ beam was then bent mechanically by moving the pushing rod to break the Au junction, which led to the molecules to adhere chemically on the fracture surface. A chamber was then evacuated to a pressure around 10^−6^ Torr so as to remove the solvent. Thereafter, stretching of the junction was implemented by a feedback control of the piezo-actuator expansion. Specifically, the strain rate was set to 1 nm/s during elongation of Au nanocontacts until *G* decreased to below 6 G_0_. After that, the junction elongation speed was set to be 0.5 pm/s by applying a *V*_p_ ramp of 4 mV/s. This slow straining yielded enough amount of time for precise measurements of the junction thermopower and conductance. When *G* eventually dropped to lower than 10^−5^ *G*_0_, Au electrodes were reconnected. The series of breaking/connecting processes were repeated for 200 times at each *V*_h_ condition from 2.0 V to 5.0 V.

### Thermoelectric voltage and electrical conductance measurements

The electrical conductance and thermovoltage of junctions were measured simultaneously during the tensile stretching. For this, a constant dc bias voltage *V*_h_ was applied to a Pt microheater throughout the experiments to create a temperature differential at the junctions necessary to induce detectable amount off thermovoltage. Furthermore, a picoammeter/source unit (Keithley 6487) was used to apply the dc voltage *V*_b_ = 0.1 V and acquire the current flowed through the junctions, from which the conductance *G* = *I/V*_b_ was obtained (the deduction included subtraction of a serial resistance). After the acquisition of *G, V*_b_ was set to zero and the potential drop *V*_m_ at the thermovoltage-sensing resistor having variable resistance *R*_s_ from 10 kΩ to 1 MΩ was obtained using a nanovoltmeter (Keithley 2182). *R*_s_ was changed electrically during the junction breaking in response to *G* by using a home-built relay device. The junction thermoelectric voltage Δ*V*_j_ was computed from *V*_m_ considering the division at *R*_s_ and 1/*G*. Subsequently, *V*_b_ was switched back to 0.1 V for the next conductance measurement. The sampling rate of *G* and *V*_m_ was about 3 Hz.

### First-principle calculations

Gaussian 09 package was employed to characterize mechanical stretching effects on molecular junction configurations. The atomic motifs of a single-molecule junction were modelled by respectively placing BDA, or ABT between two Au14 clusters. Density functional theory (DFT) level of calculations were performed with B3LYP hybrid functional for optimizing the junction structures. The basis sets of 6–31 G + (d, p) and LANL2DZ were employed for (C, H, S, N) and (Au), respectively. Completing the optimization processes, the distance between the two molecule-linking Au atoms was widened gradually by 0.2 Å steps. At every each step, the structural optimization was performed and the bond length as well as the angle at the metal-molecule links were measured from the coordination of the atoms. The series of calculations were continued until the molecules bridged the Au clusters.

### Theoretical derivation of quadruple molecular junction strucutre

To deduce the parallel bridging structure of multiple molecules in an electrode gap, we started modelling from an optimized structure of a single-molecule junction wherein ABT is placed between two Au14 clusters. In the optimized geometry, N-C and S-C bonds of the ABT molecule leaned to 97.4° and 99.7° from [111] direction of each Au14 cluster. By expanding a Au14-ABT-Au14 motif to Au22-ABT-Au22 structure, we constructed the slab model of 3 × 3 supercell and then conducted geometry and cell optimization calculations by using a density functional based tight binding (DFTB) method with the AuOrg Slater-Koster parameters under a periodic boundary condition. All calculations were performed by using Material Studio package. The optimized lattice parameters were a = b = 8.02 Å and c = 42.9 Å, α = 118°, β = 117° and γ = 61.0°, and the atomic distance of Au for molecular transport and direct tunnelling was evaluated as 9.52 Å and 6.29 Å, respectively^55^,^56^.

## Additional Information

**How to cite this article:** Tsutsui, M. *et al*. Roles of vacuum tunnelling and contact mechanics in single-molecule thermopower. *Sci. Rep.* **7**, 44276; doi: 10.1038/srep44276 (2017).

**Publisher's note:** Springer Nature remains neutral with regard to jurisdictional claims in published maps and institutional affiliations.

## Supplementary Material

Supplementary Information
