# Supplementary material for: Roles of vacuum tunnelling and contact mechanics in single-molecule thermopower
Source: Sci Rep. 2017 Mar 10;7:44276. doi: 10.1038/srep44276 (PMC5345045; doi:10.1038/srep44276)
Supplement: Supplementary Information [file srep44276-s1.pdf]

Supplementary Information for

Roles of vacuum tunnelling and contact mechanics in single-molecule thermopower

Makusu Tsutsui, Kazumichi Yokota, Takanori Morikawa & Masateru Taniguchi

The Supplementary Information includes:

1. Supplementary Figures (Figures S1 – S9)

2. Supplementary references

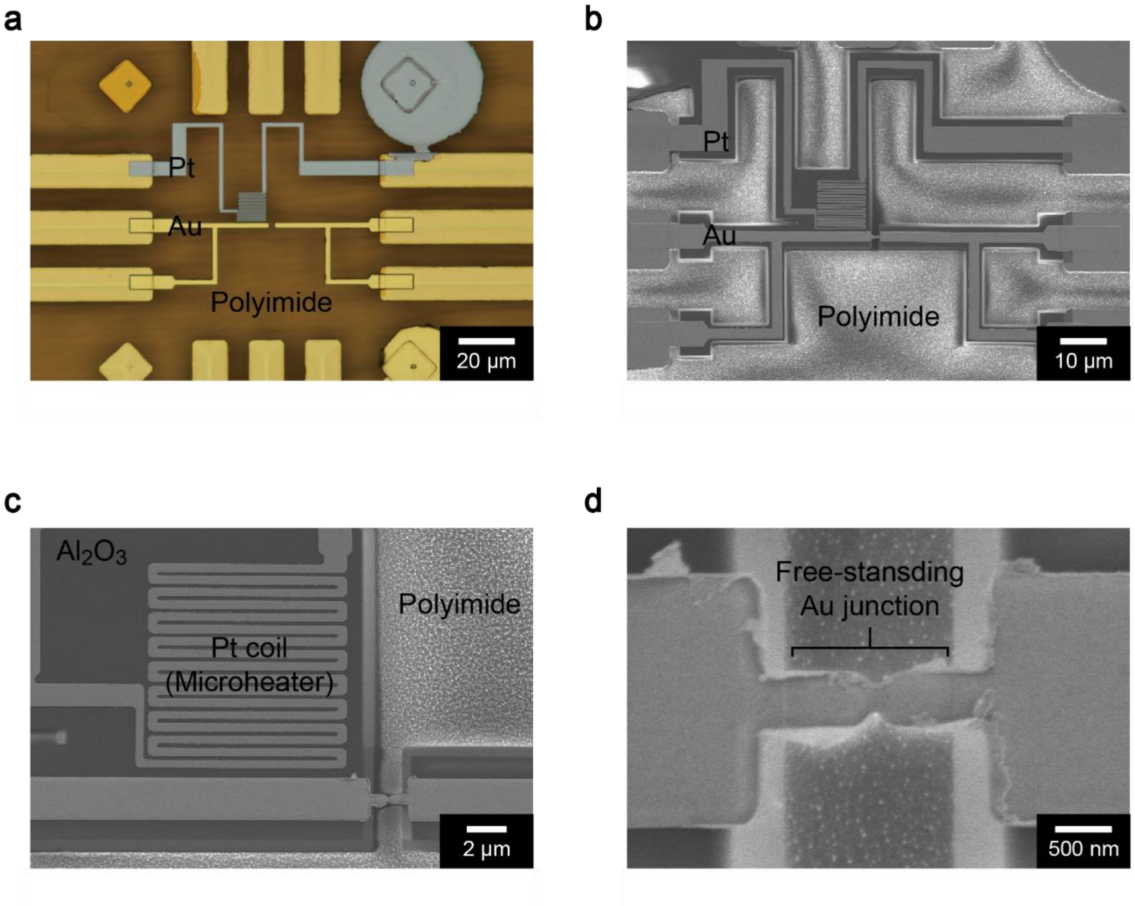

Figure S1. Microheater-embedded mechanically-controllable break junctions (MCBJs). **a-b,** Optical (a) and scanning electron micrographs (b) of a microheater-embedded MCBJ. **c-d,** Close views of a Pt coil microheater (c) and a free-standing Au junction (d). The Al_2O_3 layer serves as a thermal link to the Au junction for creating a temperature differential via Joule heating at the Pt heater. The whole structure was fabricated on a thick polyimide functioning as both electrical and thermal insulator layer to suppress leakage of electrical and heat current to the substrate. The heat control performance of the device is reported elsewhere [S1,S2].

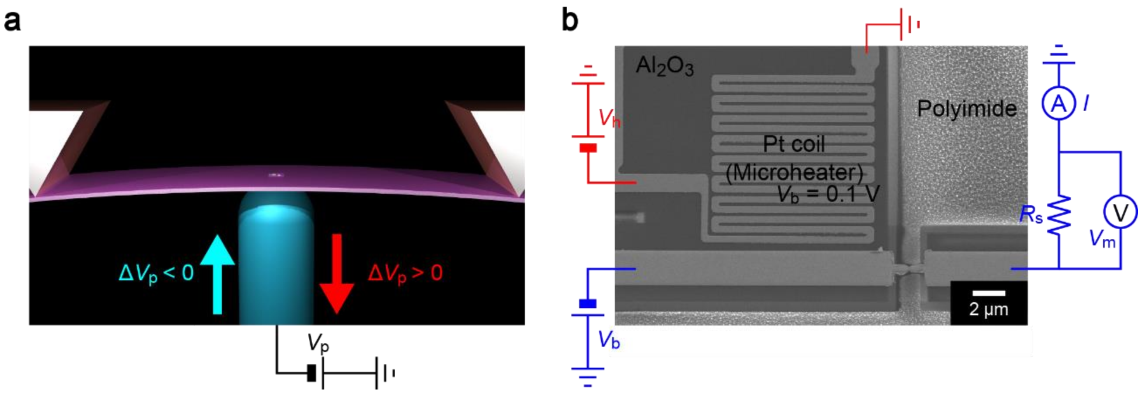

Figure S2. MCBJ mechanism and measurement circuit. **a,** Schematic model explaining the piezo-control of the MCBJ substrate deflection. The piezo-actuator moves the rod to push or retract the bending beam in response to a change in the applied voltage ΔV_p by 1 um/ΔV_p. **b,** Measurement circuit used to perform simultaneous acquisitions of the junction conductance and the thermovoltage. The voltage V_h was applied to the Pt coil microheaterr for generating a temperature gradient along the Au junctions. Meanwhile, another voltage V_b = 0.1 V was imposed to the Au junction and the output current I was recorded, from which the junction conductance G was obtained through $G = I / V_{eff}$, where V_{eff} is the actual potential drop occurred at the junction that considers division at the serial resistor R_s. After the single G measurement, V_b was set to zero and the voltage drop at R_s was measured using a nanovoltmeter for deducing the junction thermopower. Then, V_b was set back to 0.1 V for G measurement. In this way, we could perform the simultaneous thermovoltage and conductance measurements at a sampling rate of 3 Hz.

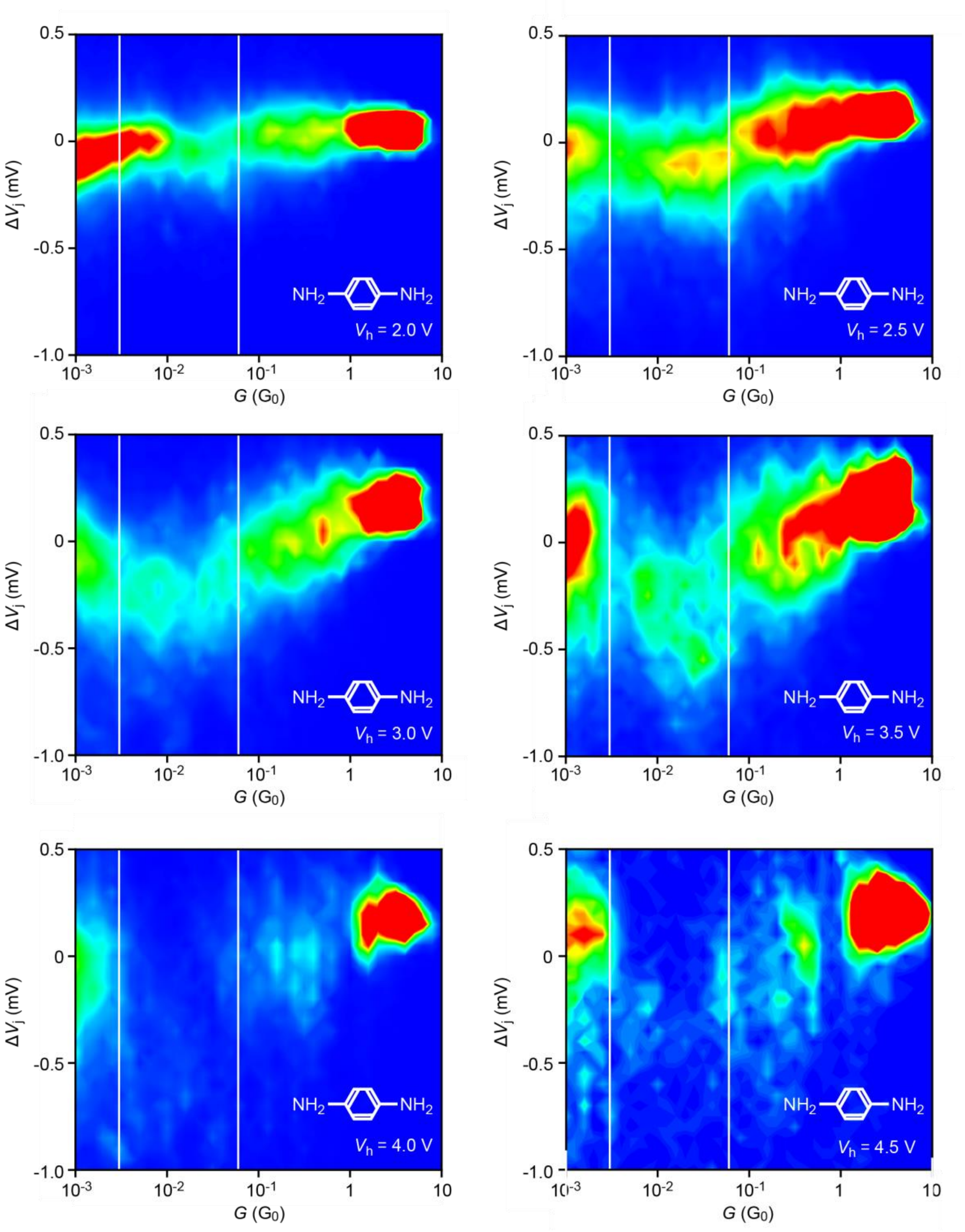

Figure S3. Conductance versus thermovoltage two dimensional histograms of 1,4-benzenediamine (BDA) molecular junctions. The positive thermoelectric voltage at the conductance G larger than 1 G_0 denotes the negative thermopower of Au atom-sized contacts [S1,S3]. White lines indicate the conductance regime where formations of Au-BDA-Au junctions is

anticipated as marked by the negative thermovoltage reflecting the HOMO-mediated electron transmission in BDA bridges.

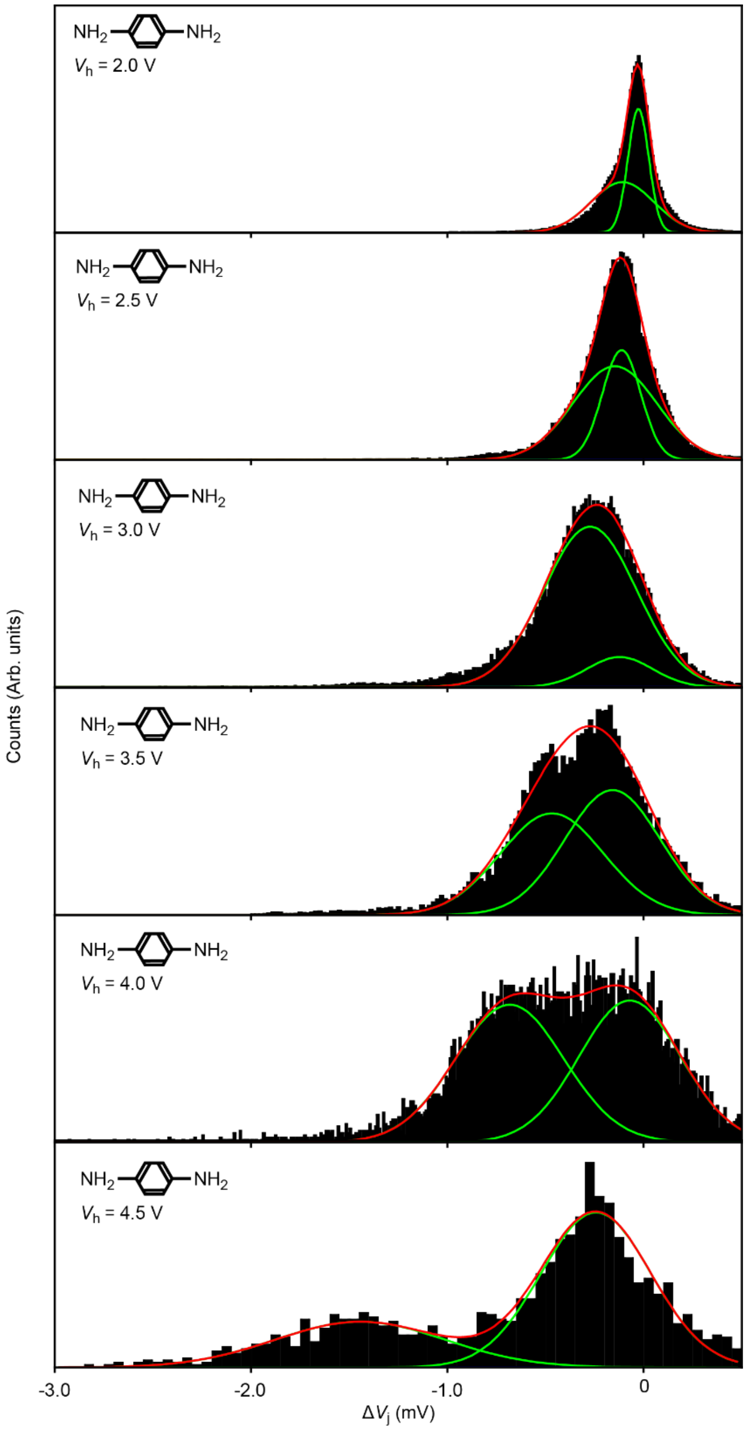

Figure S4. Thermovoltage distributions of 1,4-benzenediamine molecular junctions. Solid curves are the Gaussian fit to the bimodal distributions. Red curve indicate the summation of the three Gaussian distributions.

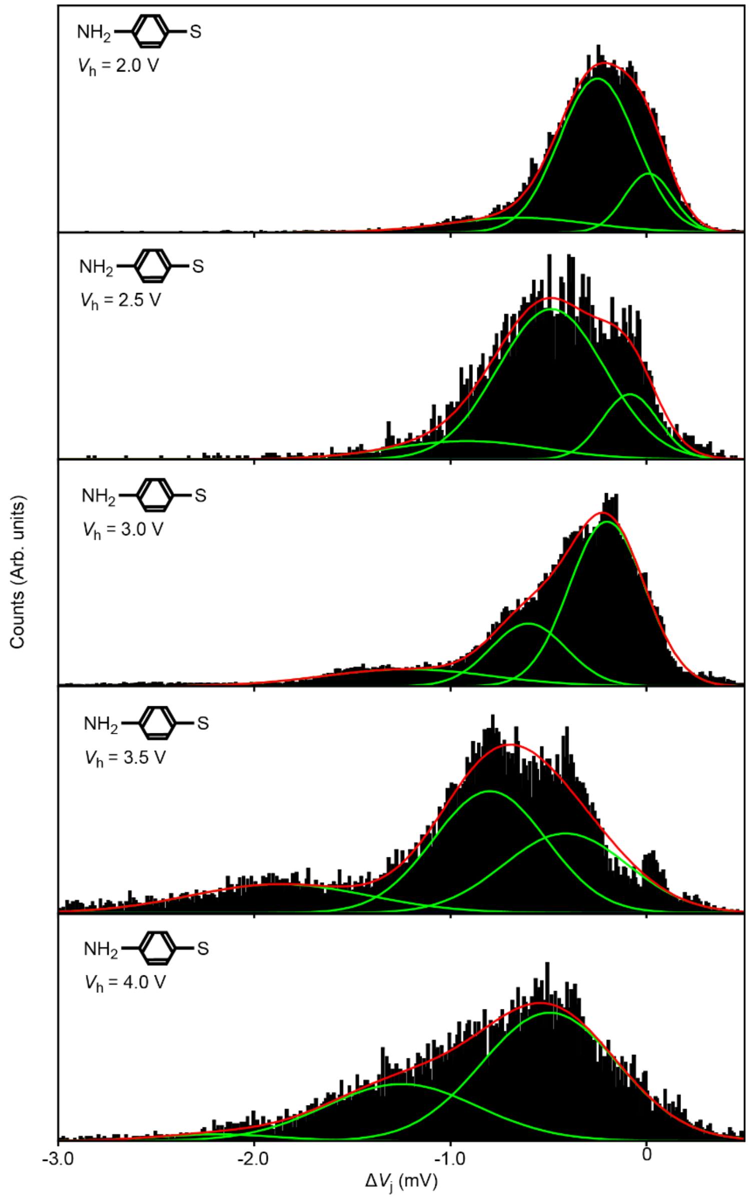

Figure S5. Thermovoltage distributions of aminobenzenethiol molecular junctions. Solid green curves are the Gaussian fit to the trimodal distributions. Red curve indicate the summation of the three Gaussian distributions.

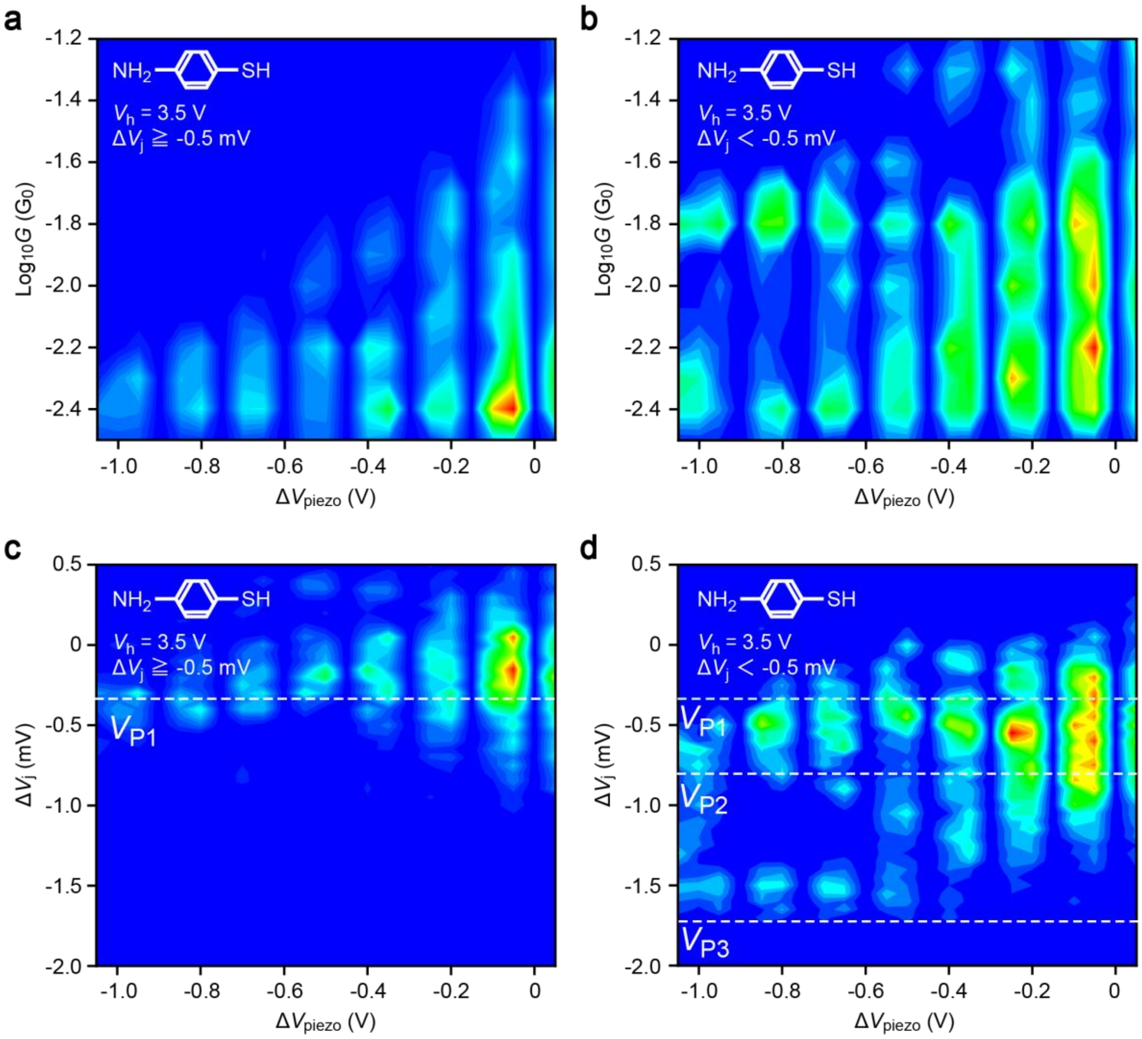

Figure S6. Junction diagnosis for aminobenzenethiol (ABT) molecular bridges. a-b, Conductance versus piezo-voltage two-dimensional histograms for traces showing the thermovoltage higher (a) or lower (b) than - 0.5 mV on average, respectively. The exponential decay in the conductance *G* in (a) suggests electron tunneling through a vacuum gap, which in turn implies absence of molecules between the Au electrodes. On the other hand, plateau-like feature in (b) signifies formations of molecular junctions. **c-d,** The corresponding thermovoltage heat maps showing the low (c) and high (d) thermoelectric voltage states ascribable to that of a vacuum gap and the ABT junctions, respectively as discussed in the main text.

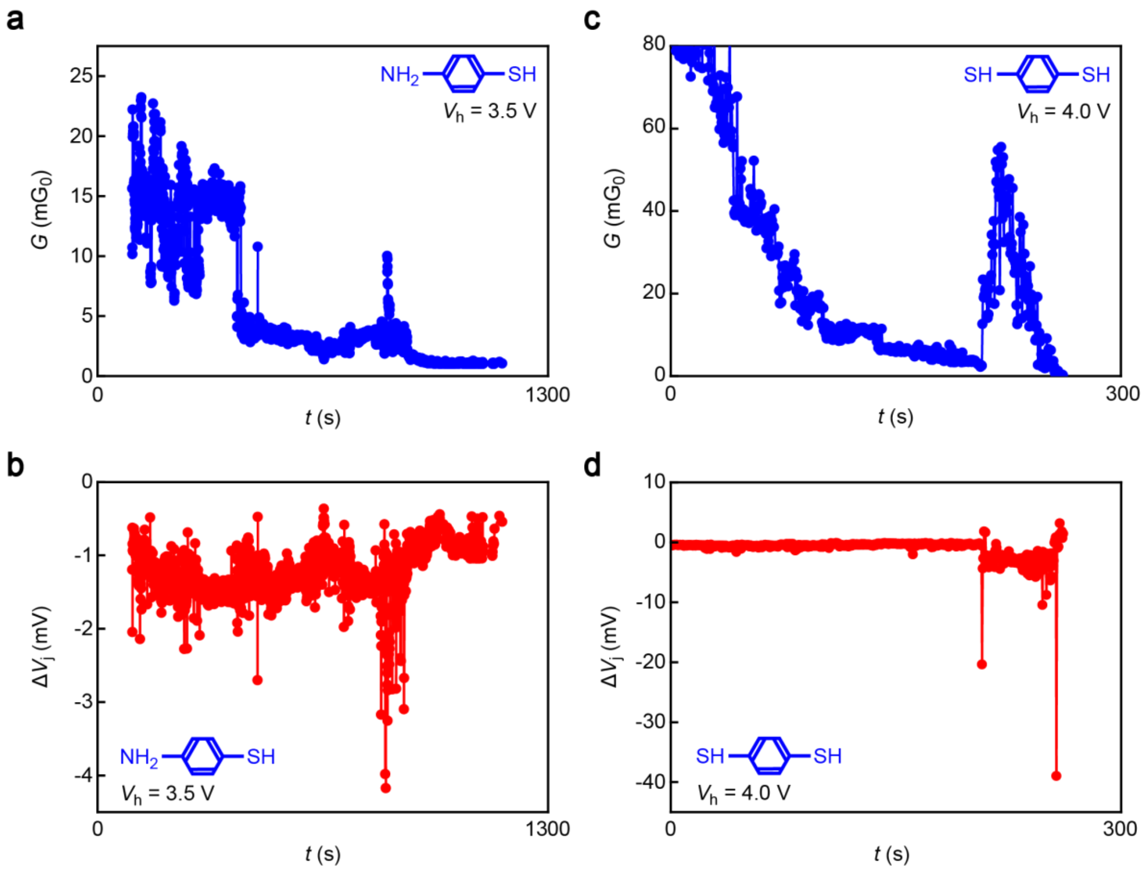

Figure S7. High thermopower of stretched molecular junctions. a-b, Conductance (a) and thermovoltage (b) traces of an ABT junction showing concomitant rapid increase in the thermoelectric properties upon stretched to undergo contact breakdown. **c-d,** The mechanically-induced thermovoltage enhancement observed in BDT junctions, which can be attributed to a modification of the length and angle of the Au-S bond [S2,S4,S5].

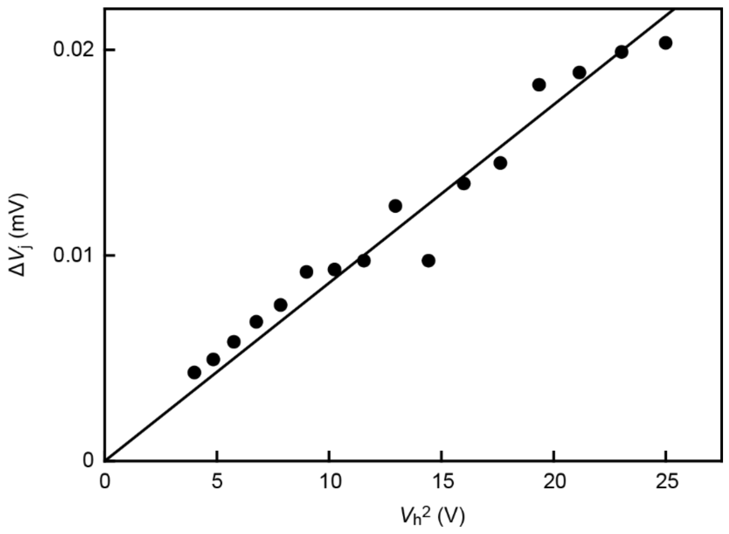

Figure S8. Estimations of temperature difference at molecular junctions. Plots of the average thermovoltage ΔV_{Au} of Au nanocontacts as a function of second power of the heater voltage V_h. The solid line is a least squares fitting. The slope α_{Au} defines the thermopower of Au contacts S_{Au} as $S_{Au} = -\alpha_{Au}/\beta$ where β is the ratio between the temperature difference ΔT and V_h^2; hence $\Delta T = \beta V_h^2$. Here, $S_{Au} = S_q - S_{bulk}$ where S_q = -0.75 μV/K and S_{bulk} = 1.96μV/K for ballistic Au atom-sized contacts and the bulk Au, respectively. For each device used to measure the thermoelectric properties of BDT, BDA, and ABT, we obtained β of 3.2, 4.9, and 6.9, respectively. ΔT was deduced from these β values.

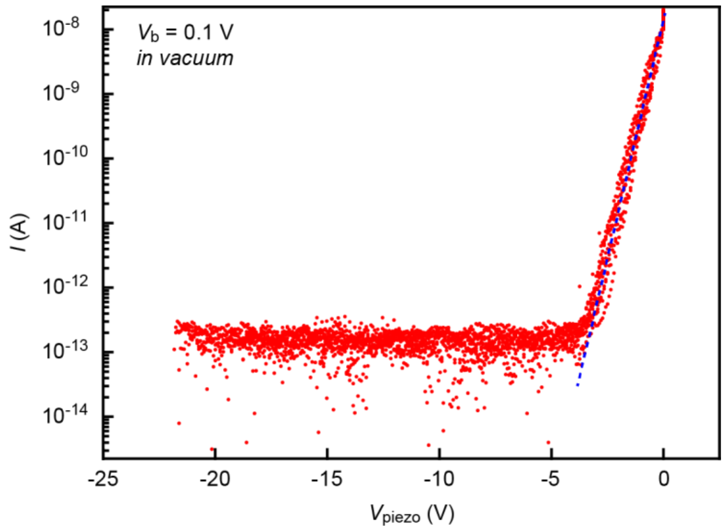

Figure S9. Calibration of junction elongation. Semi-logarithmic plots of the tunnelling current I as a function of the piezo-voltage V_{piezo} during gradual closure of a Au electrode gap at room temperature in vacuum under zero heater voltage. Blue line is an exponential fit at the V_{piezo} regime whereat the tunneling current showed rapid increase with the interelectrode distance Δd. The piezo-actuator moves by – 1 μm/V whereby pushing and bending an MCBJ substrate. We examined the measurements ten times by opening/closing the contact. The results were overplotted in the figure, revealing little scattering in the data suggesting reliable and stable control of the gap distance. The slope γ was used to deduce the rate of change in Δd by V_{piezo} through $I \propto \exp(-2\kappa\Delta d) = \exp(\gamma V_{piezo})$, and hence $\gamma V_{piezo} = -2\kappa\Delta d$ with the tunneling decay constant $\kappa = \sqrt{2m_e\Phi/\hbar^2}$ where $m_e = 9.11 \times 10^{-31}$ kg is the electron mass. This yielded the displacement ratio d_{gap}/V_{piezo} of 1.3×10^{-10} m/V.

2. Supplementary references

S1. Tsutsui, M.; Morikawa, T.; Arima, A. & Taniguchi, M. Thermoelectricity in atom-sized junctions at room temperatures. *Sci. Rep.* **3**, 3326 (2013).

S2. Tsutsui, M.; Morikawa, T.; He, Y.; Arima, A. & Taniguchi, M. High thermopower of mechanically stretched single-molecule junctions. *Sci. Rep.* **5**, 11519 (2015).

S3. Evangeli, C.; Matt, M.; Rincon-Garcia, L.; Pauly, F.; Nielaba, P.; Rubio-Bollinger, G.; Cuevas, J. C. & Agrait, N. Quantum thermopower of metallic atomic-size contacts at room temperature. *Nano Lett.* **15**, 1006 (2015).

S4. Andrews, D. Q.; Cohen, R.; van Duyne, R. P. & Ratner, M. A. Single molecule electron transport junctions: Charging and geometric effects on conductance. *J. Chem. Phys.* **125**, 174718 (2006).

S5. Saffarzadeh, A.; Demir, F. & Kirczenow, G. Mechanism of the enhanced conductance of a molecular junction under tensile stress. *Phys. Rev. B* **89**, 045431 (2014).
